# Dr. Charles McMillan

**Published:** 1890-02

**Authors:** 


Dr. Charles McMillan.—We regret to chronicle the death of Dr. Charles McMillan, the Medical Referee of the Pension Bureau, which occurred in Washington, D. C., January 7, 1890, of pneumonia.

Dr. McMillan entered the volunteer service of the army August 3, 1861, and served until October 7, 1865, when he was mustered out. He served as American Consul at Rome during General Grant's terms as president.

